# Modeling Chronic BaP Exposure in Bronchial Epithelial Cells Reveals Multi-Scale Drivers of Early Preneoplastic Reprogramming

**DOI:** 10.3390/cells15060566

**Published:** 2026-03-22

**Authors:** Cristian Andrade-Madrigal, Cecilia Rojas-Fuentes, Javier Díaz-Mijares, Gloria M. Calaf, Pablo M. Santoro, Alejandro H. Corvalán, Francisca J. Medina, Cristian G. Torres, Paula Romero-Vicencio, Julio C. Tapia, Mónica L. Acevedo, Ricardo Soto-Rifo, Enrique Boccardo, Francisco Aguayo

**Affiliations:** 1 Laboratory of Oncovirology, Departamento de Ciencias Biomédicas, Facultad de Medicina, Universidad de Tarapacá, Arica 1000000, Chile; cristian.andradem@gmail.com; 2 Laboratorio de Virología Molecular y Celular, Núcleo Interdisciplinario de Microbiología, Instituto de Ciencias Biomédicas (ICBM), Facultad de Medicina, Universidad de Chile, Santiago 8380453, Chile; cecrojas@uchile.cl (C.R.-F.); diazm7@correo.uss.cl (J.D.-M.); monica.acevedo@uchile.cl (M.L.A.); rsotorifo@uchile.cl (R.S.-R.); 3 Instituto Milenio en Inmunología e Inmunoterapia, Santiago 8380453, Chile; 4 Center for HIV/AIDS Integral Research (CHAIR), Facultad de Medicina, Universidad de Chile, Santiago 8380453, Chile; 5 Instituto de Alta Investigación, Universidad de Tarapacá, Arica 1000000, Chile; gmcalaf@academicos.uta.cl; 6 Department of Hematology and Oncology, Faculty of Medicine, Pontificia Universidad Católica de Chile, Portugal 61, Santiago 8330023, Chile; p.santoro.v@gmail.com (P.M.S.); acorvalan@uc.cl (A.H.C.); 7 Departamento de Ciencias Clínicas, Facultad de Ciencias Veterinarias y Pecuarias, Universidad de Chile, Santiago 8320000, Chile; francisca.medina@veterinaria.uchile.cl (F.J.M.); crtorres@uchile.cl (C.G.T.); 8 Laboratorio de Transformación Celular, Núcleo Interdisciplinario de Biología y Genética (NiBG), Instituto de Ciencias Biomédicas (ICBM), Facultad de Medicina, Universidad de Chile, Av. Independencia 1027, Santiago 8380453, Chile; paula.romero.1@ug.uchile.cl (P.R.-V.); jtapiapineda@uchile.cl (J.C.T.); 9 Laboratory of Oncovirology, Department of Microbiology, Instituto de Ciências Biomédicas, Universidade de São Paulo, Sao Paulo 05508-900, Brazil; eboccardo@usp.br

**Keywords:** lung carcinogenesis, benzo[a]pyrene, BPDE, chronic exposure, DDR, ATM, γ-H2AX, transcriptomics, phospho-arrays, BEAS-2B cells, ALI

## Abstract

Chronic exposure to benzo[a]pyrene (BaP), a Group 1 IARC carcinogen, is a major driver of lung carcinogenesis; however, how sustained subcytotoxic exposure reprograms bronchial epithelium toward preneoplastic states remains poorly defined. Here, we subjected BEAS-2B human bronchial epithelial cells to 12 weeks of continuous BaP at environmentally relevant concentrations (0.1 and 1.0 µM) and interrogated the resulting phenotypes using an integrated multi-scale framework encompassing functional toxicology, RT-qPCR, RNA-seq, phospho-kinase/NF-κB arrays, and organotypic air–liquid interface (ALI) cultures. Cells maintained metabolic competence throughout, evidenced by sustained CYP1A1 and CYP1B1 induction at both acute (4 h) and chronic (12-week) timepoints, while accumulating genotoxic stress as demonstrated by dose-dependent nuclear γ-H2AX foci formation and ATM phosphorylation (Ser1981). RNA-seq revealed a dose-dependent transcriptional shift: 0.1 µM BaP yielded 119 differentially expressed genes (DEGs; |log2FC| ≥ 1, FDR < 0.05), whereas 1.0 µM generated 255 DEGs. Downregulated transcripts were enriched for extracellular matrix and cell-adhesion programs (COL14A1, ADAMTS2, CSMD3, CADM3), while upregulated genes encompassed inflammatory, calcium-signaling, and vesicle-trafficking modules (NFATC4, CSF2RA, SYT1, PCLO). Phospho-kinase/NF-κB arrays confirmed a p53/NF-κB signaling nexus, with concurrent activation of MAPK/ERK (Thr202/Tyr204) and PI3K/Akt (Ser473) pathways. Despite persistent genotoxic stress, cells did not acquire anchorage-independent growth and remained non-tumorigenic in vivo. Critically, ALI organotypic cultures derived from BaP-exposed cells exhibited histological dysplasia, nuclear pleomorphism, and disrupted apical-basal polarity. These findings mechanistically link chronic BaP exposure to an initiation-like preneoplastic state and establish a validated 2D/3D multi-omics platform for PAH-driven lung carcinogenesis research.

## 1. Introduction

Lung cancer remains the leading cause of cancer-related mortality worldwide [1,2], with its etiology tightly linked to chronic exposure to environmental carcinogens and tobacco-derived pollutants [3]. In most real-world settings, these exposures occur as complex polycyclic aromatic hydrocarbon (PAH) mixtures in tobacco smoke and urban particulate matter [4,5]. Among these, benzo[a]pyrene (BaP), a prototypical PAH [6], is classified as a Group 1 carcinogen by the International Agency for Research on Cancer (IARC) due to its potent mutagenic activity following metabolic activation into BaP-7,8-diol-9,10-epoxide (BPDE) [7]. BaP robustly engages the aryl hydrocarbon receptor (AhR) transcriptional program (including CYP1A1/CYP1B1 induction) and intersects with inflammatory and pro-survival signaling. Together, these effects suggest that chronic exposure selects for damage-tolerant epithelial states, rather than producing simple acute cytotoxicity [8]. Despite the genotoxic properties of BaP, particularly the formation of BPDE-DNA adducts and mutation induction, are well established [9,10], carcinogenesis is now understood as a multistep evolutionary process that extends beyond DNA damage alone [11,12]. Accordingly, early “preneoplastic” transitions may involve sustained stress responses, altered differentiation/adhesion, and signaling rewiring that precede overt transformation but bias the trajectory toward malignancy. In this study, a “preneoplastic state” is operationally defined by the concurrent fulfillment of three criteria: (i) acquisition of molecular hallmarks of carcinogen-driven re-programming, including persistent DNA damage response (γ-H2AX, p-ATM), dose-dependent transcriptional remodeling (DEGs by RNA-seq), and activation of pro-survival and inflammatory signaling networks (phospho-kinase arrays); (ii) emergence of tissue-level dysplastic features in organotypic ALI cultures (nuclear pleo-morphism, loss of apical-basal polarity, aberrant proliferative compartmentalization); and (iii) absence of full malignant transformation, as evidenced by lack of anchor-age-independent growth and failure to form tumors in immunodeficient mice. This definition is explicitly multi-scale and functional and avoids equating the preneoplastic state with any single molecular or histological marker [13,14].

Yet the experimental models used to study these transitions have not kept pace with this conceptual shift. Conventional in vitro toxicology has relied predominantly on acute, high-dose exposures in two-dimensional (2D) monolayer cultures [15,16]. While informative for cytotoxicity and early genotoxic events, such models fail to capture the adaptive and compensatory responses that emerge during chronic exposure [17]. Thus, new methodologies have been increasingly adopted to improve human relevance in toxicological research. Organotypic air–liquid interface (ALI) cultures represent a particularly powerful methodology for respiratory toxicology, as they recapitulate key aspects of airway epithelial organization, polarity, and cell–cell interactions under exposure scenarios [18,19,20,21].

The immortalized human bronchial epithelial cell line BEAS-2B has been widely used to study xenobiotic metabolism and carcinogen-induced transformation due to its metabolic competence and capacity for long-term culture. It should be noted, however, that BEAS-2B cells are immortalized via the Ad12-SV40 hybrid virus, whose large T antigen (LTAg) can interact with and partially sequester p53, potentially attenuating canonical p53-mediated responses relative to primary bronchial epithelial cells [22]. While this does not preclude the use of BEAS-2B cells as a model for chronic carcinogen exposure [23], they retain xenobiotic-metabolizing capacity [24], AhR signaling [25], and DDR activation, it does impose an important interpretative boundary: conclusions regarding p53 as a central regulatory hub must be contextualized within the partial p53 constraint inherent to this cell line. Culture under standard serum-containing (FBS) two-dimensional conditions also confers a partial epithelial–mesenchymal phenotype on BEAS-2B cells, as previously documented [26], which should be considered when interpreting transcriptional changes associated with ECM and adhesion programs. These limitations, and their implications for translating findings to primary cell models, are discussed in detail in Section 4. However, a systems-level understanding of how chronic BaP exposure reshapes BEAS-2B cells across molecular, signaling, and tissue scales remains incomplete [27]. It remains unclear how sustained metabolic activation and DNA damage induced by BaP intersect with transcriptional and signaling network remodeling to drive early preneoplastic epithelial states [25,28]. Notably, most experimental models of BaP toxicity rely on acute high-dose exposure, which poorly reflects the chronic low-dose exposure scenarios characteristic of environmental and tobacco-related carcinogenesis.

Here, we aimed to define how chronic BaP exposure remodels bronchial epithelial cells across multiple biological scales. Using an integrated multi-omics and multi-scale functional framework, we combined viability and metabolic assays, transcriptomic profiling, phosphoproteomic signaling analysis, and organotypic air–liquid interface (ALI) cultures. This approach reveals coordinated metabolic, transcriptional, and signaling remodeling that functionally links chronic BaP exposure to early epithelial reprogramming and preneoplastic progression. Our findings establish a human-relevant experimental framework for studying long-term carcinogen effects and demonstrate that ALI cultures generated from BEAS-2B cells after chronic BaP exposure provide a robust platform for mechanistic respiratory toxicology.

## 2. Materials and Methods

### 2.1. Cell Culture

The human bronchial epithelial cell line BEAS-2B (CRL-3588, American Type Culture Collection (ATCC), Manassas, VA, USA) was utilized in this study. These are non-tumorigenic cells, originally isolated from bronchial tissue biopsies and immortalized via the Ad12-SV40 hybrid virus [29]. BEAS-2B cells were maintained at 37 °C in a humidified atmosphere with 5% CO_2_ using DMEM (SH30243.02, HyClone (Cytiva), Logan, UT, USA) supplemented with 10% FBS (SV30160.03, HyClone (Cytiva), Logan, UT, USA) and 1X Antibiotic-Antimycotic Solution (30-004-CI Corning, Corning, NY, USA), providing a final concentration of 100 units/mL penicillin G, 0.1 mg/mL streptomycin sulfate, and 0.25 µg/mL amphotericin B. Routine passaging was performed using TrypLE Express (12604-021, Gibco, Waltham, MA, USA). For experiments requiring precise cell quantification for seeding, Trypsin-EDTA (25-053-CI, Corning, Corning, NY, USA) was used instead. In both procedures, 700 µL of the dissociation reagent was applied per 25 cm^2^ flask, followed by incubation at 37 °C for 2–3 min. The cells were then resuspended in the supplemented culture medium. Green’s Differentiation Medium: Comprising a 3:1 mixture of DMEM and F12, supplemented with 10% FBS, 1X Antibiotic-Antimycotic Solution, 0.02 nM cholera toxin (C8052; Sigma-Aldrich, St. Louis, MO, USA), 5 g/mL apo-transferrin (T-1147, Sigma-Aldrich, St. Louis, MO, USA), 0.4 g/mL hydrocortisone (H-4881, Sigma-Aldrich, St. Louis, MO, USA), 5 g/mL insulin (I-1882, Sigma-Aldrich, St. Louis, MO, USA), and 10 ng/mL human Epidermal Growth Factor recombinant protein (hEGF) (AF-100-15, PeproTech, Cranbury, NJ, USA).

### 2.2. Chronic Benzo[a]pyrene Exposure

A 100 mM stock solution of BaP (B1760, Sigma-Aldrich, St. Louis, MO, USA) was prepared in DMSO (D1370.1000, Duchefa Biochemie, Haarlem, The Netherlands). For the dose–response assays, serial dilutions were performed in DMSO and supplemented culture medium, maintaining a constant final DMSO concentration of 0.01% (*v*/*v*) across all conditions. Based on the preliminary results, concentrations of 0.1 μM and 1.0 μM BaP were selected for the 12-week chronic treatment. Accordingly, 10,000X stock solutions (i.e., 1000 μM and 10,000 μM, respectively) were prepared in DMSO. Chronic exposure was maintained for 12 weeks, with cells being passaged twice weekly (every 3 to 4 days). Throughout the experimental period, the culture medium was supplemented with 0.1 μM BaP, 1.0 μM BaP, or 0.01% (*v*/*v*) DMSO as a vehicle control.

### 2.3. Cell Viability Assay

To determine the optimal seeding density for subsequent experiments, cells were seeded in 96-well plates at densities ranging from 500 to 3000 cells per well. Cell proliferation was evaluated using the CellTiter 96^®^ AQueous One Solution Cell Proliferation Assay (G3580, Promega, Madison, WI, USA) (MTS) according to the manufacturer’s instructions and our protocols [30]. Briefly, the supplemented culture medium was removed and replaced with 100 µL of serum-free DMEM. Subsequently, 20 µL of the MTS reagent was added to each well, followed by incubation for 1 h at 37 °C. After a brief mechanical agitation, absorbance was quantified at 492 nm using a Synergy HTX multi-mode microplate reader (BioTek Instruments, Winooski, VT, USA).

### 2.4. Quantitative Real-Time PCR (RT-qPCR)

Total RNA was extracted using the E.Z.N.A. Total RNA Kit I (R6834-4000CH, Omega Bio-tek, Norcross, GA, USA) following the manufacturer’s protocol. As specified in the optional instructions, β-Mercaptoetanol (1610710, Bio-Rad, Hercules, CA, USA) was added to the lysis buffer. All centrifugation steps were performed at 20,000× *g* at room temperature. To ensure the complete removal of residual ethanol carryover, an additional dry-spin step was included by centrifuging the columns at 21,000× *g* for 1 min in a clean microcentrifuge tube prior to elution. Subsequently, 1 μg of total RNA was treated with RQ1 RNase-Free DNase (M610A, Promega, Madison, WI, USA) to eliminate genomic DNA contamination. Complementary DNA (cDNA) synthesis was then performed using MMLV Reverse Transcriptase (M1705, Promega, Madison, WI, USA), utilizing oligo(dT) primers (C110A, Promega, Madison, WI, USA), dNTPs (U1515, Promega, Madison, WI, USA) and RNAsin Ribonuclease Inhibitor (N251A, Promega, Madison, WI, USA), according to the manufacturer’s instructions. The resulting cDNA was subjected to qPCR analysis using the SensiFAST SYBR No-ROX Kit (BIO-98020, Bioline (Meridian Bioscience), London, UK). Reactions were carried out in a final volume of 10 μL, containing 1 μL of cDNA, 0.2 μL of each 10 μM primer, and the master mix. The amplification was performed according to the thermal cycling conditions established by the manufacturer. Primer sequences for RT-qPCR used in this study are shown in Supplementary Table S1. In each RT-qPCR run, no-reverse-transcriptase controls (no-RT) and no-template controls (NTCs) were included to verify the absence of genomic DNA contamination and reagent/template contamination, respectively. No amplification was detected in any no-RT or NTC reaction throughout the study.

### 2.5. RNA Sequencing and Analysis

Following two months of chronic exposure to BaP, cryopreserved cells were thawed and maintained for an additional month as described above, completing a total exposure period of three months. RNA sequencing was performed after prior validation of BaP-induced transcriptional changes by RT-qPCR. Total RNA was extracted as detailed in Section 2.4, with an input of 5,000,000 cells per column. RNA concentration and purity were assessed by absorbance ratios on a NanoDrop One spectrophotometer (Thermo Fisher Scientific, Waltham, MA, USA). Library preparation was performed using the MGI platform with the following kits: the MGIEasy rRNA Depletion Kit (MGI, Shenzhen, China) for ribosomal RNA removal, the MGIEasy RNA Library Prep Kit (MGI, Shenzhen, China) for non-directional library construction, and the MGIEasy Circularization Module V2.0 (MGI, Shenzhen, China). MGI adapter sequences are provided in Supplementary Table S1. Sequencing was performed in paired-end 150 bp (PE150) mode on a DNBSEQ-G400 sequencer using the DNBSEQ-G400RS High-throughput Sequencing Set (MGI). Raw reads were (paired-end FASTQ files) were first subjected to quality control (QC) using FastQC version 0.11.9 to assess per-base sequence quality, GC content, sequence duplication levels, and adapter contamination. As an initial pre-processing step, reads were screened with Bowtie2 version 2.5.1 for removal of contaminant/rRNA-mapping reads. To remove adapter sequences and low-quality bases, reads were trimmed using Trim Galore version 0.6.4 (which leverages Cutadapt). Reads shorter than a minimum length threshold after trimming were discarded, and additional fixed-length trimming with fastq_trimmer of Fastx-toolkit version 0.0.13 was applied when required to standardize read length across libraries. The cleaned reads were then aligned to the Human Reference Genome (GRCh38.p14) using BWA-MEM version 0.7.17: 23, r1188. Gene-level read counts were computed using featureCounts from Rsubread package version 2.14.2 (part of Bioconductor version 3.17) by assigning aligned fragments to annotated genomic features (gene models) to generate a sample-by-gene count matrix. Differential expression analysis was performed in R using DESeq2 version 1.40.2, which models raw count data using negative binomial generalized linear models and applies internal normalization (size factors) to account for library depth. Resulting *p*-values were corrected for multiple testing using the Benjamini–Hochberg procedure, and genes were considered differentially expressed based on an FDR-adjusted *p*-value threshold (FDR < 0.05) and an effect-size cutoff (log2FC ≥ 1 or log2FC ≤ −1). Functional enrichment analysis was performed using Gene Ontology (GO release 2025-10-10) annotations restricted to the Biological Process category, conducted separately for upregulated and downregulated gene sets using a hypergeometric test; enriched terms were considered significant at FDR-adjusted *p* < 0.05. Results were visualized as dot plots in which dot size represents gene count and color intensity reflects statistical significance. To identify upstream transcriptional regulators, transcription factor (TF) network analysis was performed using the TRRUST v2 database [31,32], mapping differentially expressed genes to experimentally validated TF–target interactions to infer key regulatory hubs and integrate transcriptomic changes with downstream phosphoproteomic analyses.

### 2.6. Phospho Kinase Array Analysis

Cells were thawed as described in Section 2.5, completing a total of three months of chronic BaP exposure. The Proteome Profiler Human Phospho-Kinase Array (ARY003C, R&D Systems, Minneapolis, MN, USA) and the Proteome Profiler Human NF-κB Pathway Array (ARY029, R&D Systems, Minneapolis, MN, USA) are antibody-based membrane arrays in which target proteins are captured by spotted antibodies and detected via biotinylated detection antibodies and streptavidin-conjugated horseradish peroxidase, generating a chemiluminescent signal proportional to target phosphorylation levels. Total protein was extracted following the manufacturer’s instructions for each array. Protein concentration was quantified using the Pierce BCA Protein Assay Kit (23227, Thermo Scientific, Waltham, MA, USA) [30]. For each condition, 225 µg of total protein lysate was loaded onto each membrane set and processed according to the manufacturer’s protocol, with the exception that chemiluminescence detection was performed using Clarity Max Western ECL Substrate (1705062, Bio-Rad, Hercules, CA, USA). Digital images were captured using the ChemiDoc Imaging System (Bio-Rad, Hercules, CA, USA). Integrated pixel density was quantified using Fiji [33] (ImageJ, version 1.54p; National Institutes of Health), and all values were normalized to the corresponding dot blot signals from DMSO-treated control cells. Both independent replicates (n = 2) showed consistent directionality of phosphorylation changes across all detected targets, supporting the reliability of the observed patterns.

### 2.7. Immunofluorescence Analysis

Cells (1 × 10^5^ viable cells per well) were seeded onto 12 mm round glass coverslips, previously sterilized by autoclaving, in 12-well plates. After overnight incubation at 37 °C and 5% CO_2_, the cells were washed twice with PBS and fixed for 20 min with 4% formaldehyde in PBS, prepared from methanol-free Pierce 16% Formaldehyde (*w*/*v*) (28906, Pierce Biotechnology, Thermo Fisher Scientific, Rockford, IL, USA). Following PBS washes, the cells were incubated with 0.1 M glycine (1610718, Bio-Rad, Hercules, CA, USA) in PBS for 10 min to quench unreacted aldehydes. Subsequently, the cells were washed three times with PBS and permeabilized with 0.2% Triton X-100 (Sigma-Aldrich, St. Louis, MO, USA) in PBS for 5 min. After two additional PBS washes, non-specific binding was blocked using 5% BSA (A1391,0100, PanReac AppliChem, Darmstadt, Germany) in PBS. Primary antibodies, diluted in blocking solution, were then added and incubated at 37 °C in a humidified chamber. Following two PBS washes, the cells were incubated with secondary antibodies under the same conditions as the primary antibodies. After two final PBS washes, the coverslips were briefly rinsed by dipping into sterile ultrapure water (3 s) and allowed to air-dry (cell-side up). The coverslips were then mounted onto glass slides using 30 µL of ProLong Diamond Antifade Mountant with DAPI (P36966, Molecular Probes, Life Technologies, Eugene, OR, USA). The slides were allowed to cure for 24 h at room temperature in the dark and subsequently stored at 4 °C until analysis by confocal fluorescence microscopy. The primary antibodies used for immunofluorescence included mouse anti-ATM (cat. no. ab78, Abcam, Cambridge, UK; 1:200), mouse anti-P-ATM (cat. no. ab36810, Abcam, Cambridge, UK; 1:200), and mouse anti-P-H2A.X (cat. no. 05-636, Merck, Darmstadt, Germany; 1:100). Secondary detection was performed using donkey anti-mouse Alexa Fluor 594 (cat. no. A-21203, Invitrogen, Waltham, MA, USA) and donkey anti-rabbit Alexa Fluor 647 (cat. no. A-31573, Invitrogen, Waltham, MA, USA), both at a final concentration of 5 µg/mL. The reading was made in a C2 Plus confocal microscope (Nikon Instruments Inc., Tokyo, Japan). In each staining experiment, secondary-antibody-only controls (omitting the primary antibody) were included in parallel and confirmed negligible background fluorescence, validating the specificity of the signals observed for p-ATM, total ATM, and γ-H2AX.

### 2.8. Organotypic Air–Liquid Interface (ALI) Cultures

To develop a stratified epithelium, BEAS-2B cells were induced to differentiate using the organotypic culture method originally described by Green [34]. For these experiments, BEAS-2B cells chronically exposed to 0.01% DMSO (vehicle control), 0.1 µM BaP, or 1.0 µM BaP for 12 weeks were used as the source of cells for ALI induction. A fibroblast-embedded collagen matrix was first prepared by combining Rat Tail Type I Collagen (354236, Corning, NY, USA), reconstitution buffer, F12 medium (21700075, Gibco, Waltham, MA, USA), and irradiated 3T3-J2 mouse fibroblasts (CVCL_W667). Collagen-fibroblast matrices were allowed to polymerize at room temperature, then equilibrated with supplemented BEAS-2B culture medium. The following day, BEAS-2B cells were seeded onto each collagen-fibroblast matrix in BEAS-2B culture medium, supplemented with an equal volume of Green’s Differentiation Medium. Twenty-four hours after seeding, the collagen–fibroblast matrices were detached with a sterile spatula and transferred onto a 2 × 2 cm sterile stainless-steel mesh placed in a 60 mm dish [35]. Differentiation medium was added until it reached the level of the mesh without overflowing, thereby establishing the air–liquid interface. Raft cultures were maintained at 37 °C with 5% CO_2_ for 10 days, with medium replaced every two days.

### 2.9. Histology and Immunohistochemistry of ALI Cultures

Raft cultures were carefully excised from stainless-steel meshes, fixed in 4% paraformaldehyde, and embedded in paraffin. Serial sections (4–5 µm thick) were prepared for histological and immunohistochemical analyses. For general assessment of epithelial architecture, sections were stained with hematoxylin and eosin (H&E) following standard protocols. For immunohistochemistry (IHC), paraffin sections were deparaffinized, rehydrated, and subjected to heat-induced antigen retrieval using citrate-based buffer. Endogenous peroxidase activity was quenched, and nonspecific binding was blocked prior to incubation with primary antibodies. Sections were incubated with antibodies against pan-cytokeratin (Pan-CK) (cat. no. sc-8018; Santa Cruz Biotechnology, Dallas, TX, USA), and Proliferating Cell Nuclear Antigen (PCNA) (cat. no. sc-56; Santa Cruz Biotechnology, Dallas, TX, USA), followed by appropriate HRP-conjugated secondary antibodies from VECTASTAIN Elite ABC-HRP Kit, Peroxidase (Mouse IgG) (cat. no. PK-6102, Vector Laboratories, Newark, CA, USA). Immunoreactivity was visualized using a chromogenic substrate, and sections were counterstained with hematoxylin. Slides were examined by light microscopy to evaluate epithelial stratification, nuclear morphology, proliferation patterns, and marker localization. Representative images from at least three independent ALI cultures per condition were captured using a CX31 microscope (Olympus Corporation, Tokyo, Japan) equipped with an ICC50 W microscope camera (Leica Microsystems, Wetzlar, Germany).

### 2.10. Clonogenic Assay

This procedure was carried out according to previously published protocols [36]. Briefly, cells were detached using Trypsin-EDTA, quantified, and seeded at a low density of 500 cells per 60 mm dish. After a 10-day incubation period, the medium was removed, and the plates were washed with PBS. The resulting colonies were fixed with ice-cold methanol for 15 min and subsequently stained with a 0.5% (*w*/*v*) crystal violet (Winkler, Santiago, Chile) solution (prepared in 20% methanol (Merck, Darmstadt, Germany) and 80% sterile ultrapure water) for 5 min. The plates were then carefully rinsed with PBS to remove excess dye and allowed to air-dry. Finally, the colonies were quantified.

### 2.11. Anchorage Independent Growth Analysis

To assess malignant transformation, a soft agar colony formation assay was performed using BEAS-2B cells that had been chronically exposed to 0.01% DMSO, 0.1 µM BaP, or 1.0 µM BaP for 12 weeks, following protocols previously published [37]. Briefly, a 0.7% (*w*/*v*) base agar layer (Winkler, Santiago, Chile) was prepared by adding 2 mL of nutrient agar per well in 6-well plates. The plates were allowed to solidify overnight within a Class II biosafety cabinet. Subsequently, a top layer was prepared by mixing 400 µL of 0.7% nutrient agar with 200 µL of a cell suspension containing 200 viable cells in supplemented DMEM. To maintain hydration and nutrient levels, 500 µL of supplemented DMEM was added to each well once a week. Grown colonies were stained by 0.05% Cristal violet after 3 weeks of incubation at 37 °C and 5%CO_2_.

### 2.12. Animal Model

A short-term pilot xenograft assay was performed in immunodeficient mice to evaluate whether chronic BaP exposure confers overt tumorigenic capacity to BEAS-2B cells. Female NOD/SCID gamma mice (3–4 weeks old) were obtained and maintained at the Bioterio Central (Faculty of Medicine, University of Chile) under controlled temperature (21–23 °C) and light/dark cycling (~12 h), with food and water provided ad libitum. BEAS-2B cells chronically exposed for 12 weeks to vehicle (0.01% DMSO) or to 1.0 µM BaP were harvested, washed, and resuspended in sterile PBS. Mice were assigned to receive a single subcutaneous injection in the flank containing ~1–2 × 10^6^ viable cells in 0.1–0.2 mL using a tuberculin syringe. At day 30 post-inoculation, animals were anesthetized with ketamine/xylazine (100/10 mg/kg) (Laboratorio Richmond División Veterinaria, B1615ICH Grand Bourg, Buenos Aires, Argentina) and euthanized by cervical dislocation. All procedures were conducted under institutional animal-care oversight and according to the approved protocol (CBA 1216 FMUCH).

### 2.13. Statistical Analysis

Statistical analyses were conducted using GraphPad Prism software version 9.5.1. A repeated measures two-way ANOVA followed by Šídák’s multiple comparisons test was used to assess differences in cell viability and RT-qPCR data, where the experimental design included two independent variables (time and treatment). Colony formation and soft-agar assays involved a limited number of independent replicates; therefore, these data were analyzed using the non-parametric Kruskal–Wallis test followed by Dunn’s multiple comparisons test, without assuming a normal distribution. Statistical significance was set at *p* < 0.05 (*), *p* < 0.01 (**), *p* < 0.001 (***), and *p* < 0.0001 (****).

## 3. Results

### 3.1. Chronic Low-Dose BaP Exposure Induces Metabolic Adaptation Without Compromising Cell Viability

**Figure 1:**
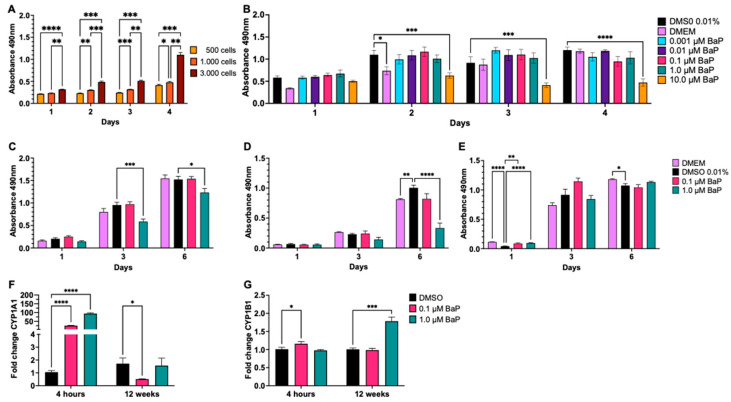
Viability of BEAS-2B epithelial cells chronically treated with benzo[a]pyrene (BaP). Proliferation assay determined cell quantity per well by measuring absorbance at 490 nm. (**A**) Dose–response assay setup; (**B**) Acute (1–4 days) dose–response assay with BaP concentrations from 0.1 µM to 10 µM, DMEM (supplemented culture medium), or vehicle control 0.01% DMSO (dimethyl sulfoxide). (**C**–**E**) Chronic exposure of BEAS-2B cells to DMEM (supplemented culture medium), 0.01% DMSO, 0.1 µM, or 1 µM BaP was assessed for (**C**) 4 weeks (1 month), (**D**) 8 weeks (2 months), and (**E**) 12 weeks (3 months). Viability was evaluated at 1, 3, and 6 days after each treatment; (**F**,**G**) BaP functional activity in BEAS-2B epithelial cells. Relative transcript levels of CYP1A1 and CYP1B1 were quantified after BaP treatment for 4 h (0 weeks) or 12 weeks. Fold change values were normalized to GAPDH and expressed relative to cells exposed to 0.01% DMSO. All data are presented as mean ± standard deviation (SD) (n = 3). Repeated measures two-way ANOVA test, followed by Šídák’s multiple comparison test, analyzed significant differences. Statistical significance was set at *p*-value < 0.05, with *p* < 0.05 (*), *p* < 0.01 (**), *p* < 0.001 (***), and *p* < 0.0001 (****).

To establish a physiologically relevant model of chronic environmental exposure, we first determined the proliferation rate of BEAS-2B cells at different seeding densities (Figure 1A). These cells exhibit a polygonal epithelial morphology when cultured in serum-free Bronchial Epithelial Cell Growth Medium (BEBM, Lonza, Basel, Switzerland). However, culture in the presence of fetal bovine serum (FBS) induces an elongated morphology and features characteristic of the epithelial–mesenchymal transition (EMT); nonetheless, they retain the capacity to undergo squamous differentiation in the presence of FBS [22,26]. Then we subsequently assessed the cytotoxic threshold of BaP acute dose–response assays conducted over 24–96 h (Figure 1B) revealed dose-dependent toxicity, with a progressive decrease in viability over time observed exclusively at 10 µM, but not at lower concentrations. Based on these results, we selected 0.1 µM and 1.0 µM BaP as sub-cytotoxic concentrations for chronic treatment. During continuous exposure for 4 weeks (Figure 1C) and 8 weeks (Figure 1D), cell viability progressively decreased at the higher dose compared with vehicle controls (DMSO). However, by 12 weeks of continuous exposure (i.e., under chronic conditions), cell viability recovered in the 1.0 µM BaP-treated cells. A core requirement of a chronic exposure assay is that treatment produces a measurable differential change, regardless of its nature, between vehicle-treated control cells and exposed cells. The results showed increases in CYP1A1 transcript levels only at acute (4 h) treatment with both 0.1 µM and 1.0 mM BaP (Figure 1F), while CYP1B1 levels augmented at either acute (4 h) or chronic (12 weeks) treatments with either 0.1- or 1.0 µM BaP, respectively (Figure 1G). These results confirm that the model retains sustained xenobiotic-metabolizing capacity, ensuring continuous generation of reactive metabolites throughout the chronic exposure period. In addition, these assays validate the functional activity of the BaP used in this study.

### 3.2. Chronic BaP Exposure Promotes DNA Damage and DDR

All mechanistic analyses in Section 3.2, Section 3.3, Section 3.4, Section 3.5, Section 3.6 and Section 3.7 were performed on BEAS-2B cells chronically exposed to both 0.1 µM and 1.0 µM BaP for 12 weeks. Immunofluorescence (Section 3.2), RNA-seq transcriptomics (Section 3.3 and Section 3.4), and phospho-kinase/NF-κB arrays (Section 3.5) were conducted on cells from both doses unless a specific dose is indicated in the text or figure. Clonogenic and soft-agar assays (Section 3.6) used cells from both doses; the in vivo pilot (Section 3.6) used 1.0 µM BaP-exposed cells only. ALI organotypic cultures (Section 3.7) were generated from both doses, with 1.0 µM showing the most pronounced histological changes.

**Figure 2:**
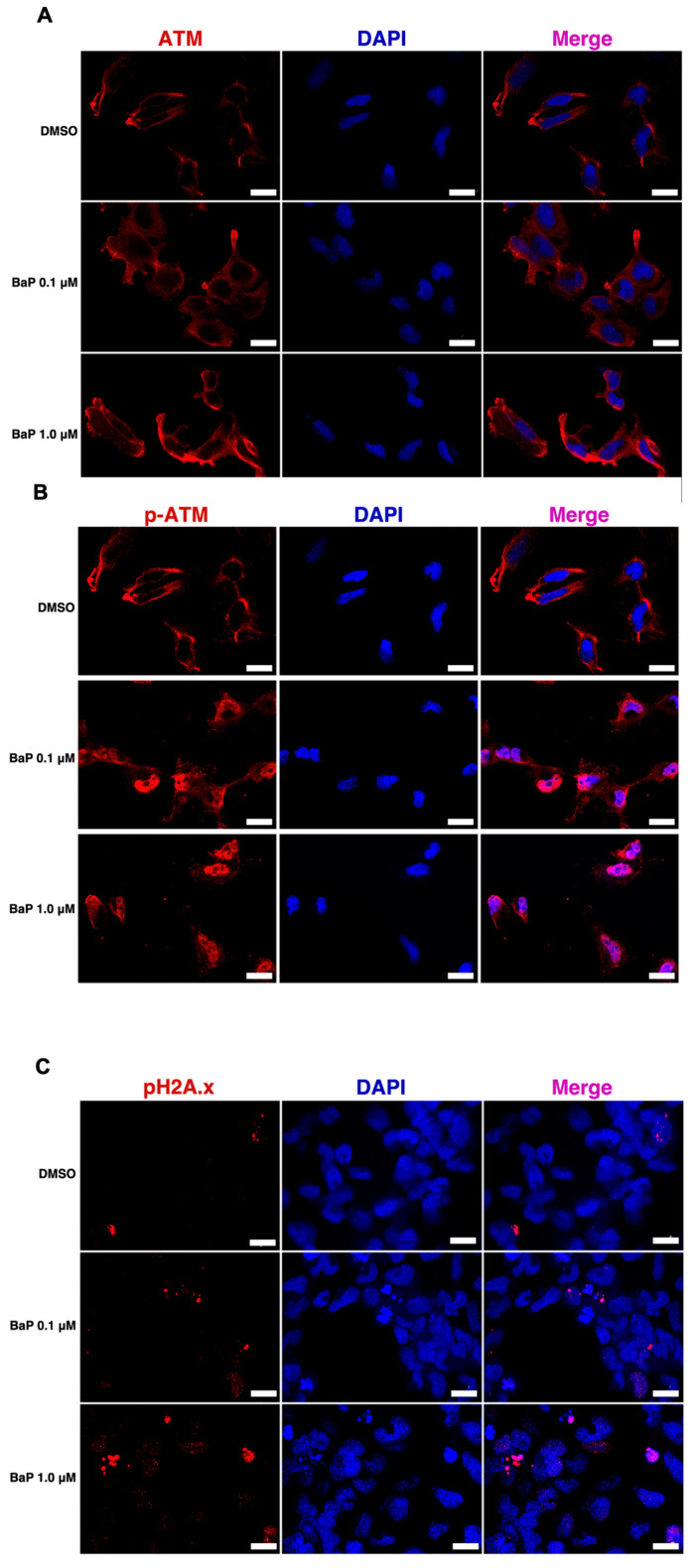
Chronic benzo[a]pyrene exposure induces dose-dependent activation of the DNA damage response in BEAS-2B cells. Representative confocal immunofluorescence images from three independent biological replicates of BEAS-2B cells chronically exposed to vehicle control (0.01% dimethyl sulfoxide, DMSO), 0.1 µM benzo[a]pyrene (BaP), or 1.0 µM BaP, showing the expression and subcellular localization of key DNA damage response (DDR) proteins. (**A**) Total ataxia–telangiectasia mutated (ATM) kinase; (**B**) Phosphorylated ATM (p-ATM, Ser1981), indicative of ATM activation and initiation of the DDR signaling cascade; (**C**) Nuclear γ-H2AX foci formation, detected as phosphorylated histone H2AX (Ser139), marking sites of DNA double-strand breaks (DSBs) and ongoing DNA repair. Red nuclear foci indicate localized DDR signaling at DNA damage sites. Scale bar = 20 µm.

We investigated whether sustained metabolic activation led to cumulative cellular damage. This genotoxic treatment triggered a DNA damage response (DDR), as evidenced by the nuclear accumulation of phosphorylated ATM (p-ATM) foci (Figure 2A,B). In addition, a dose-dependent increase in γ-H2AX was observed following BaP exposure (Figure 2C).

### 3.3. Chronic BaP Exposure Promotes Alteration of Gene Expression Associated with Tumor Progression

**Figure 3:**
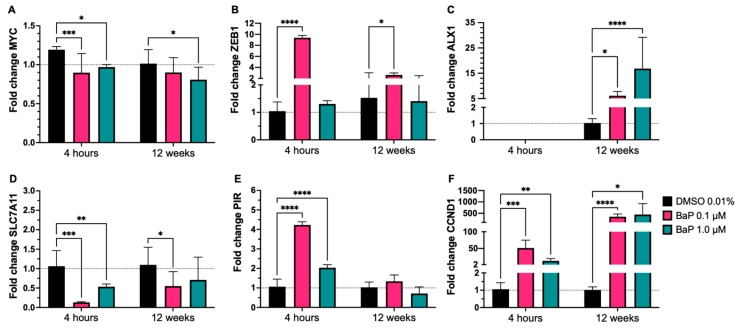
BaP alters the expression profiles of transcripts associated with EMT and the EBV replicative cycle. Fold change RT-qPCR values were normalized to GAPDH and expressed relative to cells exposed to vehicle control 0.01% DMSO (dimethyl sulfoxide) in BEAS-2B cells exposed to benzo[a]pyrene (BaP) either acutely (4 h) or chronically (12 weeks). (**A**) MYC; (**B**) ZEB1; (**C**) ALX1; (**D**) SLC7A11; (**E**) PIR; (**F**) CCND1 (Cyclin D1). Data of all figures are shown as mean ± SD (n = 3), with three technical replicates per experiment. Repeated measures two-way ANOVA test, followed by Šídák’s’ multiple comparison test, analyzed significant differences. Statistical significance was set at *p*-value < 0.05, with *p* < 0.05 (*), *p* < 0.01 (**), *p* < 0.001 (***), and *p* < 0.0001 (****).

We further evaluated whether BaP exposure under acute (4 h) and chronic (12-week) conditions is associated with changes in the expression of cellular transcripts linked to tumor progression–related traits. To this end, relative mRNA levels of MYC, ZEB1, ALX1, SLC7A11, PIR, and CCND1 (cyclin D1) were quantified by qRT-PCR in BEAS-2B cells treated with vehicle (0.01% DMSO) or BaP (0.1 µM and 1.0 µM), normalized to GAPDH, and expressed relative to vehicle-treated (0.01% DMSO) control cells (i.e., cells receiving DMSO but no BaP; no untreated, DMSO-free control was included, as the DMSO vehicle at 0.01% *v*/*v* has no detectable effect on gene expression at this concentration); data are reported as mean ± SD with statistical inference (Figure 3).

Under acute exposure (4 h), BaP elicited a modest but significant MYC reduction upon 0.1 µM BaP treatment, indicating that the early response does not reflect uniform oncogene activation but rather a selective transcriptional reprogramming (Figure 3A). Also, an early response consistent with activation of pro-progression programs, highlighted by a significant increase in ZEB1 and PIR (Figure 3B,E) (more pronounced at 0.1 µM), together with a robust induction of cyclin D1 (Figure 3F), suggested the rapid engagement of pathways related to phenotypic plasticity and cell-cycle control. In parallel, SLC7A11 decreased significantly at 4 h (particularly at 0.1 µM) (Figure 3D). After chronic exposure (12 weeks), most early changes tended to attenuate; however, a marked overexpression of cyclin D1 persisted, and a significant increase in ALX1 emerged (more evident at 1.0 µM) (Figure 3C), consistent with a late reprogramming component associated with progression-related traits. Collectively, these results indicate that BaP triggers a biphasic transcriptional response: an early induction phase involving regulators (e.g., ZEB1 and PIR) and a chronic phase in which focal alterations persist and/or arise (e.g., cyclin D1 and ALX1), which is compatible with acquisition of tumor progression–linked features.

### 3.4. Chronic BaP Exposure Induces Dose-Dependent Remodeling of the Bronchial Epithelial Transcriptome

**Figure 4:**
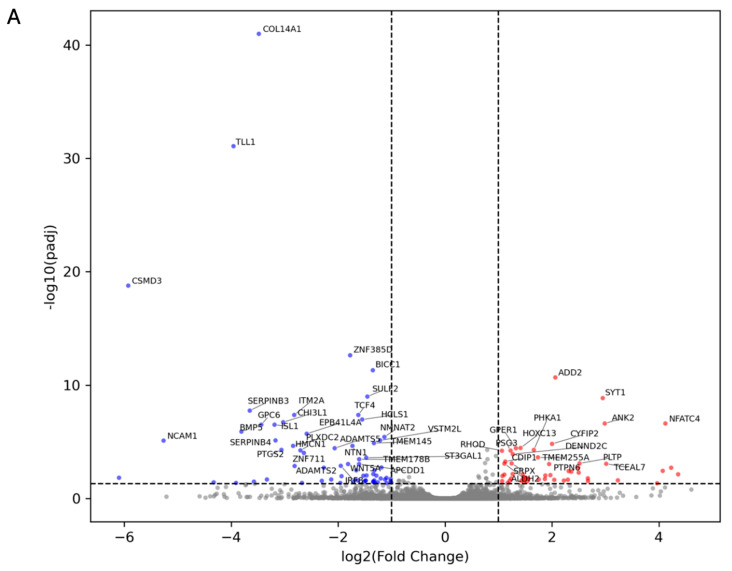

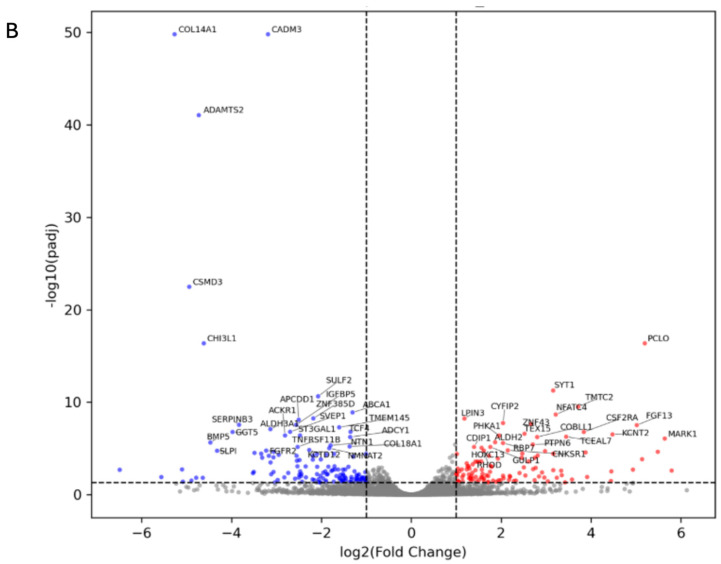
Chronic benzo[a]pyrene (BaP) exposure induces dose-dependent transcriptomic remodeling in BEAS-2B bronchial epithelial cells. Volcano plots showing differential gene expression after 12 weeks of exposure to 0.1 µM BaP (**A**) or 1.0 µM BaP (**B**) compared with vehicle control (0.01% dimethyl sulfoxide, DMSO). The x-axis represents the log_2_ fold change in gene expression, and the y-axis represents the xlog_10_ adjusted *p*-value. Differential expression analysis was performed using DESeq2 based on a negative binomial model with Wald test statistics, and *p*-values were adjusted for multiple testing using the Benjamini–Hochberg false discovery rate (FDR). Experiments were performed using three independent biological replicates (n = 3).

To characterize the baseline state of the respiratory epithelium prior to appearance of a neoplasia, we analyzed the transcriptomic profile of BEAS-2B cells exposed to low (0.1 µM) and high (1.0 µM) doses of BaP for 12 weeks. RNA-seq analysis revealed that chronic exposure elicits a specific, dose-dependent shift in gene expression rather than a broad, global dysregulation. At the lower dose (0.1 µM BaP), the response was modest, with only 51 upregulated and 68 downregulated genes (|log2FC| ≥ 1, *p* < 0.05). In contrast, increasing the concentration to 1.0 µM BaP produced a more robust reprogramming, expanding the signature to 116 upregulated and 139 downregulated transcripts. This dose-dependent amplification is clearly visualized in the volcano plots (Figure 4A,B), where the 1.0 µM condition shows a broader distribution of significantly modulated genes with larger fold changes.

**Figure 5:**
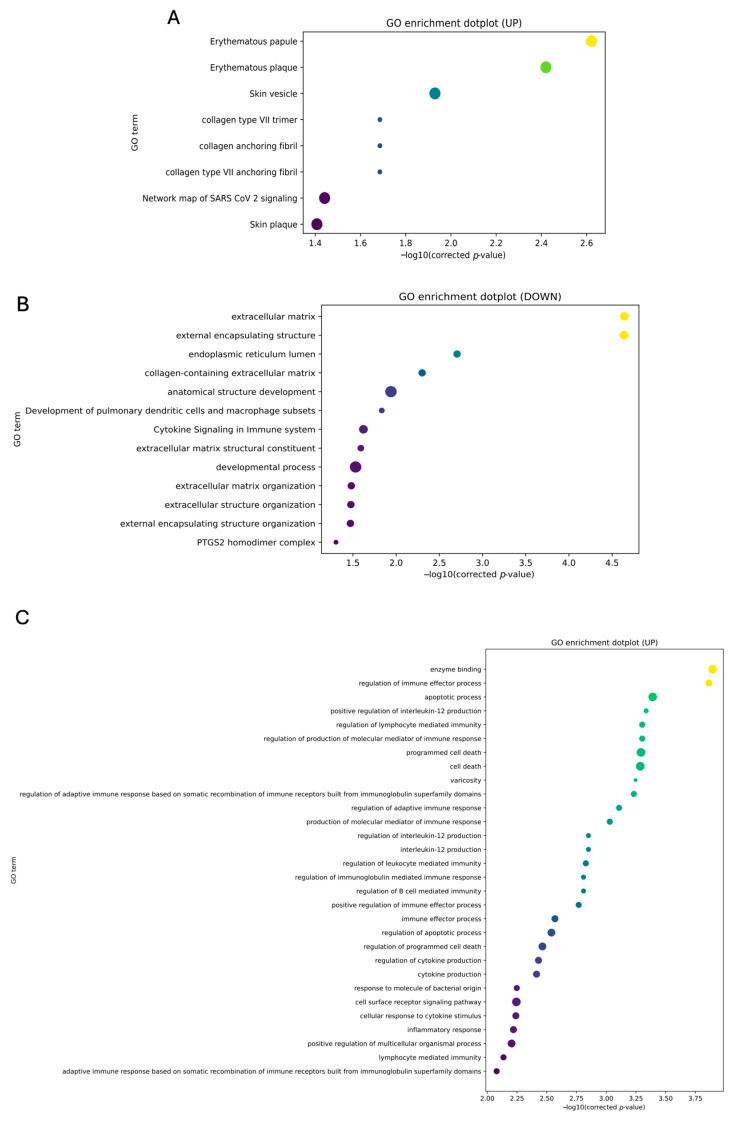

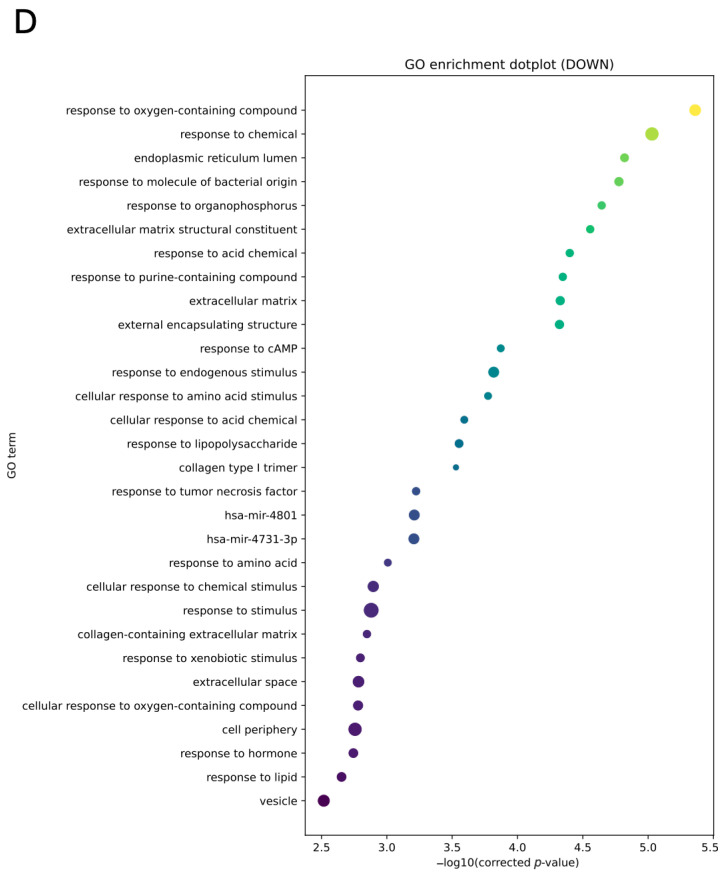
Functional enrichment of biological processes altered by BaP (Gene Ontology analysis). Dot plots of Gene Ontology (GO, release 2025-10-10) enrichment analysis restricted to the Biological Process category, performed separately for upregulated (**A**,**C**) and downregulated (**B**,**D**) differentially expressed genes in BEAS-2B bronchial epithelial cells after 12 weeks of chronic exposure to 0.1 µM BaP (**A**,**B**) or 1.0 µM BaP (**C**,**D**) relative to the vehicle control (0.01% DMSO). Differentially expressed genes were defined as |log2FC| ≥ 1 and FDR-adjusted *p* < 0.05 (Benjamini–Hochberg correction; n = 3). Enrichment significance was assessed by hypergeometric test; only GO terms with FDR-adjusted *p* < 0.05 are shown. Dot size represents the number of genes enriched in each GO term; color intensity reflects statistical significance (−log10 FDR-adjusted *p* value).

**Figure 6:**
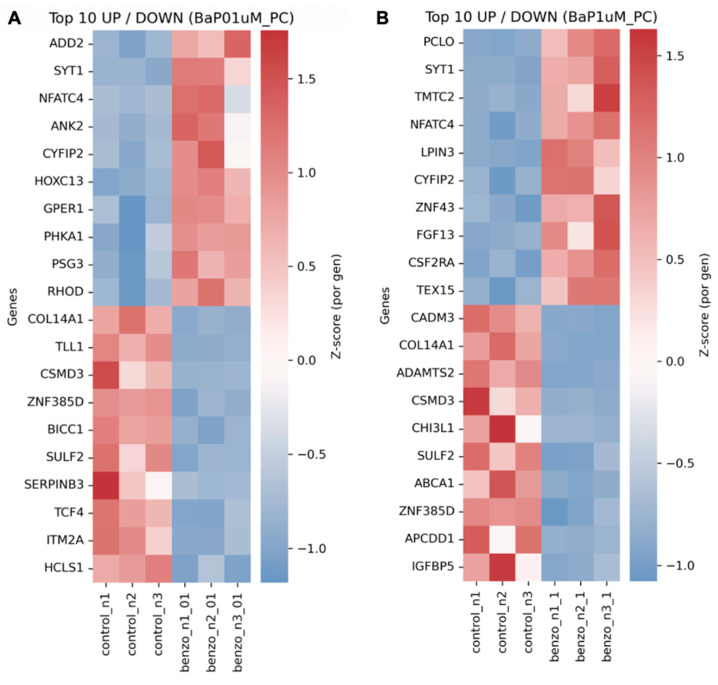
Hierarchical gene expression signatures in the transformed epithelium. Heatmaps showing the expression profiles of the top 10 upregulated and downregulated genes in cells chronically exposed to (**A**) 0.1 µM and (**B**) 1.0 µM benzo[a]pyrene (BaP) relative to vehicle control 0.01% DMSO (dimethyl sulfoxide). The color scale represents per-gene Z-scores (red: high expression; blue: low expression), highlighting key markers of extracellular matrix remodeling and immune responses (e.g., COL14A1, MMPs, ILs) identified by RNA-seq (n = 3).

In addition, BaP was found to induce a dual phenotype characterized by loss of structural integrity and activation of neuro-immune signaling (Figure 5). To identify the gene signatures driving this reprogramming, we performed hierarchical clustering of the top differentially expressed genes (Figure 6). The most significantly downregulated genes were strongly enriched of extracellular matrix (ECM) components and cell-adhesion programs, including COL14A1, ADAMTS2, CSMD3, and CADM3. In contrast, the upregulated fraction was dominated by genes involved in calcium signaling, vesicle trafficking, and inflammatory modulation, such as PCLO, SYT1, NFATC4, and CSF2RA. Notably, NFATC4 and SYT1 displayed a progressive increase in expression from 0.1 µM to 1.0 µM, supporting an accumulative, stress-driven response.

### 3.5. Transcriptional Networks and Phospho-Kinase Arrays Identify a p53/NF-κB Signaling Nexus

**Figure 7:**
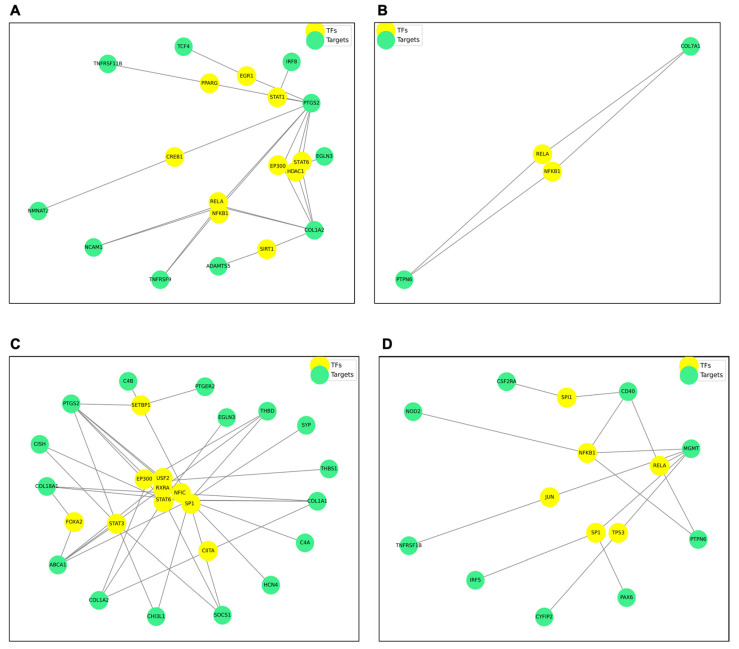
Predicted transcription factor regulatory networks associated with transcriptomic shifts following chronic benzo[a]pyrene exposure in BEAS-2B cells. TRRUST analysis was performed on differentially expressed genes (DEGs) identified by RNA-seq to map transcription factor (TF) regulatory networks. Predicted regulatory networks are shown for: TRRUST-significant downregulated genes at (**A**) 0.1 µM and (**C**) 1.0 µM BaP; and TRRUST-significant upregulated genes at (**B**) 0.1 µM and (**D**) 1.0 µM BaP (n = 3).

**Figure 8:**
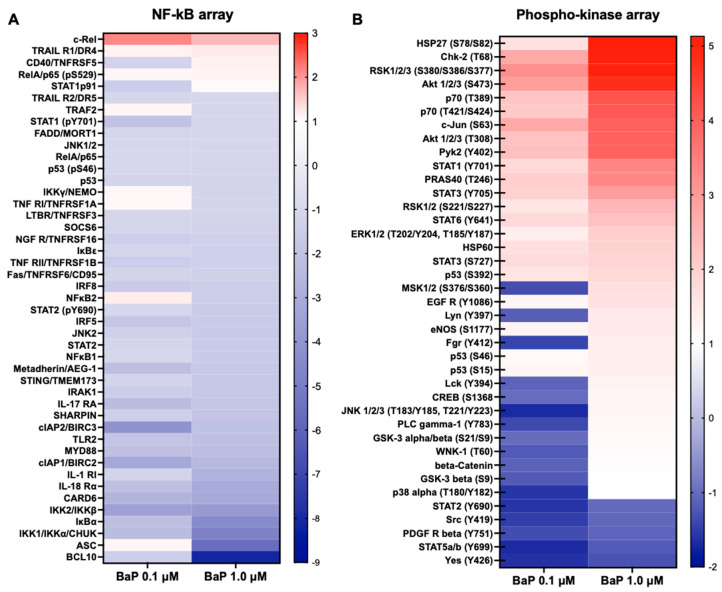
Reprogramming of cellular signaling assessed by phospho-kinase arrays/NF-κB arrays. Fold change values were normalized to protein ug and expressed relative to cells exposed to vehicle control 0.01% DMSO (dimethyl sulfoxide) in BEAS-2B cells exposed to benzo[a]pyrene (BaP) Quantitative analysis of (**A**) NF-κB pathway arrays and (**B**) phospho-kinase arrays in lysates from BEAS-2B cells chronically exposed (12 weeks) to BaP (0.1 and 1.0 µM). Heatmaps illustrate the relative phosphorylation levels of key proteins involved in survival, proliferation, and inflammation (e.g., p53, p38 MAPK, STATs, IKKs) compared with the control condition. Data indicate a dose-dependent alteration of signal-transduction networks (n = 2).

To identify the master regulators orchestrating this transcriptomic shift, we used the TRRUST database to map differentially expressed genes (DEGs) onto transcription factor (TF) regulatory networks. This analysis predicted a regulatory hub centered on NF-κB (RELA, NFKB1), AP-1 (JUN), and p53 (TP53), driving the immune and apoptotic gene signatures observed (Figure 7). We functionally validated these predictions using phosphoproteomic arrays. Consistent with the transcriptomic inference, chronically exposed cells exhibited: (i) sustained DNA damage signaling, with marked phosphorylation of p53 (Ser15/Ser46) and Chk2 (Thr68); (ii) pro-survival/inflammatory activation, evidenced by significant phosphorylation of the NF-κB subunit RelA/p65 (Ser529) and upstream regulators; and (iii) persistence of growth signals, with an unexpected hyperactivation of the PI3K/Akt (Ser473) and MAPK/ERK (Thr202/Tyr204) pathways (Figure 8).

Collectively, multi-omics integration supports that chronic BaP imposes an early state characterized by persistent genotoxic damage coupled to survival signaling and inflammatory programs, along with transcriptome-level loss of adhesion/polarity. This is consistent with the emergence of dysplastic features in the 3D ALI model and with the relationship between adhesion and epithelial organization described in this study.

### 3.6. Chronic BaP Exposure Does Not Promote Tumorigenic Transformation of BEAS-2B Cells

**Figure 9:**
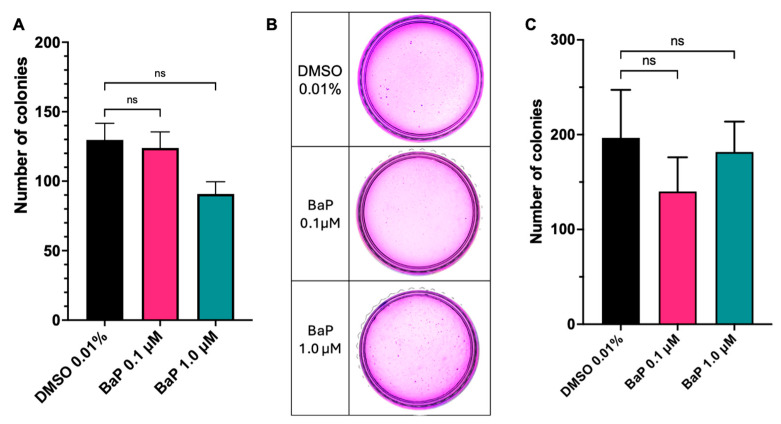
Effect of benzo[a]pyrene (BaP) on the clonogenic capacity of BEAS-2B cells. (**A**) Long-term survival and proliferative capacity were assessed using a colony formation assay. Cells were seeded at low density and treated with 0.01% DMSO (control), 0.1 µM BaP, or 1.0 µM BaP, and colonies were allowed to form over 14 days. The bar graph shows the mean number of colonies counted (>30 μm diameter) with Fiji (ImageJ) per condition, and error bars indicate the standard error of the mean (SEM) (n = 3). The Kruskal–Wallis test, followed by Dunn’s multiple comparison test, analyzed significant differences. No statistically significant differences (ns) were observed between BaP-treated groups and the control; (**B**) Anchorage-independent growth by soft-agar colony formation assay representative images; (**C**) Quantification of soft-agar colony formation assay. The bar graph shows the total number of colonies formed by BEAS-2B cells following chronic exposure to 0.01% DMSO (control) or BaP at 0.1 µM and 1.0 µM. Bars represent the mean ± standard error of the mean (SEM) (n = 3). The Kruskal–Wallis test, followed by Dunn’s multiple comparison test, analyzed significant differences. Statistical comparisons indicate no significant differences (*p* < 0.05) in colony-forming capacity relative to the control.

To assess the long-term impact of BaP exposure on reproductive viability and clonal proliferative capacity in lung epithelial cells, we performed a clonogenic assay in BEAS-2B cells. As shown in Figure 9, treatment with 0.1 µM and 1.0 µM BaP did not induce statistically significant changes in the number of colonies formed compared with the vehicle control DMSO. Although a slight downward trend in the mean colony number was observed at the higher concentration (1.0 µM BaP), colony-forming capacity remained comparable across all experimental groups under these conditions (Figure 9A). These data suggest that, at these doses, BaP does not acutely compromise the clonal survival of BEAS-2B cells.

To assess whether chronic BaP exposure could promote malignant transformation, anchorage-independent growth was evaluated using a soft-agar colony formation assay. Cells harvested from monolayer cultures after 12 weeks of chronic BaP exposure exhibited a dose-dependent change in their ability to form colonies in soft agar (Figure 9B,C). Whereas control cells and those exposed to 0.1 µM BaP formed few, small colonies, cells treated with 1.0 µM BaP did not form significantly more larger colonies. Conversely, inoculation of NOD/SCID mice with BaP-exposed BEAS-2B cells did not result in tumor formation after 30 days. Therefore, although BaP exposure elicits alterations consistent with early initiation events, these changes do not culminate in full malignant transformation of the exposed cells under the conditions tested. It should be noted that this in vivo experiment was designed as a short-term pilot assessment: the 30-day endpoint was selected as an initial screen for overt tumorigenicity, and the absence of tumor formation is interpreted as supporting, rather than definitively establishing, the preneoplastic (non-transformed) status of BaP-exposed cells.

### 3.7. Chronic BaP Exposure Promotes Dysplasia in 3D Organotypic Cultures

**Figure 10:**
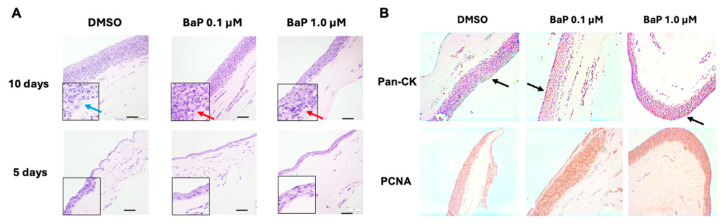
Histological and immunohistochemical characterization of organotypic cultures from BaP-chronically exposed BEAS-2B cells. (**A**) Representative hematoxylin and eosin (H&E)-stained sections of organotypic air–liquid interface (ALI) cultures after 5 and 10 days of stratification. BEAS-2B cells were chronically exposed to benzo[a]pyrene (BaP; 0.1 µM or 1.0 µM) or vehicle control (0.01% dimethyl sulfoxide, DMSO) prior to ALI induction. Images illustrate dose- and time-dependent alterations in epithelial thickness and cellular stratification. Insets show higher-magnification views of representative areas; red arrows indicate alterations in nuclear morphology and intercellular cohesion, and blue arrows indicate normal epithelial architectural organization. Scale bar = 100 µm; (**B**) Representative immunohistochemical micrographs of organotypic cultures exposed to BaP. BEAS-2B cells were chronically exposed to BaP and subsequently induced to stratify in organotypic cultures. Pan-cytokeratin (Pan-CK) staining (black arrows) was used to identify and delineate the epithelial compartment. Proliferating cell nuclear antigen (PCNA) staining indicates nuclear proliferative activity and potential replication- or DNA damage–associated responses. Experiments were performed with three independent biological replicates (n = 3).

To determine whether these molecular alterations translate into histological changes, we generated air–liquid interface (ALI) organotypic cultures from chronically exposed cells. Whereas control ALI cultures formed a stratified epithelium with organized polarity, cultures exposed to 1.0 µM BaP displayed dysplastic features (Figure 10A). Histological evaluation (H&E) revealed cellular atypia, nuclear pleomorphism, and loss of apical-basal polarity, consistent with the downregulation of adhesion-related genes observed by RNA-seq. In addition, within organotypic cultures, BaP did not abolish epithelial identity (Pan-CK+), but it did disrupt epithelial homeostasis. Specifically, at 0.1 µM BaP we observed an evident expansion of PCNA+ nuclei into suprabasal layers, compatible with a uncoupling between differentiation and cell-cycle exit (an early hyperproliferative phenotype), whereas at 1.0 µM BaP the PCNA signal decreased relative to 0.1 µM BaP, suggesting that at higher doses genotoxic stress and/or checkpoint activation predominates, thereby constraining proliferation (Figure 10B).

## 4. Discussion

This study presents the first integrated multi-scale characterization of how sustained, environmentally relevant BaP concentrations (0.1 and 1.0 µM over 12 weeks) remodel human bronchial epithelial cells at the molecular, signaling, and tissue levels, without inducing malignant conversion. Our data support an early, initiation-like adaptive phenotype rather than overt malignant conversion. Specifically, chronically exposed BEAS-2B cells preserved long-term viability while sustaining xenobiotic-metabolizing capacity, accumulated persistent genotoxic stress (ATM phosphorylation, γ-H2AX), underwent dose-dependent transcriptional remodeling (119 DEGs at 0.1 µM; 255 DEGs at 1.0 µM; |log2FC| ≥ 1, FDR < 0.05) characterized by repression of extracellular matrix/adhesion programs and induction of inflammatory and calcium/vesicle-trafficking modules, and exhibited a phospho-signaling architecture centered on a p53/NF-κB nexus with concurrent PI3K/Akt and MAPK/ERK activation. Critically, these molecular alterations translated into histological dysplasia in organotypic air–liquid interface (ALI) cultures, yet cells did not acquire anchorage-independent growth and failed to form tumors in vivo, collectively placing the observed phenotype within a preneoplastic rather than fully malignant continuum. A key feature of this model is the separation of chronic stress from acute cytotoxic collapse [38]. 

Acute dose–response experiments indicated appreciable toxicity only at 10 µM, whereas 0.1–1.0 µM permitted sustained treatment. The transient decrease in viability at 1.0 µM during weeks 4–8, followed by recovery by week 12, is compatible with progressive adaptation under continuous xenobiotic challenge. Prior chronic BaP exposure studies have employed a wide range of concentrations (typically 0.5–10 µM) [13,14], often in the absence of multi-scale functional endpoints. In the present study, the selection of 0.1 µM and 1.0 µM as sub-cytotoxic chronic concentrations was based on (i) acute dose–response viability data demonstrating that significant cyto-toxicity emerged exclusively at 10 µM (Section 3.1), and (ii) their alignment with indoor air and occupational BaP exposure levels estimated from environ-mental modeling studies [39]. These concentrations are thus situated at the lower end of the experimentally used range, permitting long-term cell survival while sustaining metabolic activation and genotoxic stress. The core innovations of the present study relative to prior chronic BaP work are: (i) the use of lower, environmentally calibrated concentrations; (ii) a 12-week exposure duration capturing adaptive transcriptional dynamics beyond early timepoints; (iii) integrated multi-omics profiling (RNA-seq + phospho-kinase/NF-κB arrays) enabling network-level mechanistic inference; and (iv) the incorporation of organotypic ALI cultures as a tissue-scale phenotypic endpoint. Importantly, sustained induction of CYP1A1 and CYP1B1 at both early (4 h) and late (12-week) time points is a critical novelty of this model: it confirms that BEAS-2B cells retain their xenobiotic-metabolizing competence throughout the entire chronic exposure period, ensuring continuous endogenous generation of the proximate carcinogen BPDE and associated genotoxic stress signaling [40,41]. This feature is absent in most prior acute-exposure models and is essential for studying carcinogen-driven preneoplastic progression under environmentally relevant conditions. Despite preserved viability, chronic exposure produced a clear genotoxic stress signature. Immunofluorescence revealed increased nuclear p-ATM (Ser1981) foci and dose-dependent induction of γH2AX [42], consistent with persistent DNA double-strand break signaling and activation of canonical DDR pathways.

While prior studies have documented BaP-associated ATM/ATR activation in acute, high-dose settings [41,43], a key scientific contribution of the present work is the demonstration that DDR signaling, evidenced by both p-ATM foci accumulation and γ-H2AX induction, is not a transient event but a sustained, chronic feature of cells exposed to environmentally relevant BaP concentrations over 12 weeks. This chronic DDR state, occurring in the context of preserved cell viability, defines a damage-tolerant cellular configuration that is fundamentally distinct from acute genotoxic responses. Phospho-signaling profiling further refined this interpretation by demonstrating activation of checkpoint and stress-response nodes. Increased phosphorylation of p53 at Ser15/Ser46 [44] and Chk2 at Thr68 [45] is consistent with ongoing DDR kinase activity [46], with p53 serving as an integrative hub that can enforce cell-cycle arrest, coordinate repair programs, and shape transcriptional remodeling [47,48]. The ALI immunohistochemistry findings align with this interpretation: PCNA staining patterns shifted with dose, with increased suprabasal PCNA positivity at 0.1 µM and reduced PCNA staining at 1.0 µM relative to 0.1 µM, consistent with stronger checkpoint engagement under higher genotoxic stress [49,50,51,52]. Together, these observations argue that chronic BaP exposure sustains DDR activation while modulating proliferative compartmentalization in a dose-dependent manner, rather than simply increasing proliferation across the board.

Targeted RT-qPCR analysis revealed a biphasic transcriptional response that distinguishes acute from chronic BaP reprogramming, a conceptually novel finding of this study. Acute exposure (4 h) induced ZEB1, PIR, and CCND1 and reduced SLC7A11 at 0.1 µM. Over 12 weeks, most acute changes attenuated, but CCND1 remained elevated and ALX1 increased, particularly at 1.0 µM. Persistent CCND1 upregulation is mechanistically significant: cyclin D1 is a convergence point for AhR, MAPK/ERK, and PI3K/Akt mitogenic signaling, and its sustained induction under chronic BaP is consistent with carcinogen-associated transformation phenotypes [49], and with the concurrent ERK activation detected in our phospho-kinase arrays. Equally important, the de novo emergence of ALX1 upregulation specifically after 12 weeks, absent at the 4 h timepoint, demonstrates that chronic BaP exposure restructures the transcriptional regulatory landscape over time, generating new steady-state programs that are not simply prolongations of acute responses [53]. This temporal dissociation between early and late transcriptional programs represents a key conceptual advance relative to acute BaP exposure models and underscores the necessity of long-term experimental paradigms for capturing carcinogen-driven regulatory reprogramming.

RNA-seq provided a systems-level view of this remodeling and showed a dose-dependent expansion of the transcriptional response [54]: 0.1 µM BaP yielded 51 upregulated and 68 downregulated genes, while 1.0 µM produced 116 upregulated and 139 downregulated transcripts (|log2FC| ≥ 1, FDR < 0.05). Gene Ontology enrichment and clustering converged on two major themes: repression of extracellular matrix and adhesion-related programs, and induction of inflammatory and calcium/vesicle-trafficking processes. Among the most downregulated transcripts were COL14A1, ADAMTS2, CSMD3, and CADM3, implicating altered extracellular and intercellular interactions. Critically, this downregulation of ECM/adhesion programs was directly mirrored by the histological findings in ALI cultures, where 1.0 µM BaP-exposed cells displayed loss of apical-basal polarity, cellular atypia, and nuclear pleomorphism, providing functional validation of the transcriptomic signatures at the tissue level. Although loss of ECM/adhesion gene expression does not by itself prove invasive behavior, the convergence of molecular and histological evidence represents a novel and rigorous demonstration of how BaP-driven transcriptional remodeling translates into tissue-scale dysplasia [55,56]. Conversely, upregulated genes such as NFATC4, SYT1, PCLO, and CSF2RA point to activation of calcium-dependent transcriptional control, vesicle/secretory trafficking, and inflammatory/immune-related signaling. The progressive, dose-dependent upregulation of NFATC4 and SYT1, increasing from 0.1 µM to 1.0 µM BaP, is a particularly novel finding, as calcium-dependent NFAT signaling and synaptic vesicle-associated programs have not been previously described as components of the BaP-driven bronchial epithelial response. These data suggest that chronic PAH stress engages non-canonical adaptive axes that may contribute to the inflammatory and secretory phenotype of early preneoplastic epithelium [57,58,59,60]. Integrating transcriptomic data with network inference and phospho-signaling profiling clarified candidate regulatory drivers. TRRUST-based transcription factor mapping nominated NF-κB (RELA/NFKB1), AP-1 (JUN), and p53 (TP53) as central hubs, and phospho-kinase arrays functionally validated the activation of both the p53 checkpoint axis and NF-κB inflammatory control. Increased phosphorylation of RelA/p65 at Ser529 and of upstream NF-κB regulatory kinases provides direct biochemical evidence that inflammatory transcriptional programs are actively engaged, rather than simply inferred from mRNA changes, representing an important technical and mechanistic advancement over RNA-only approaches. At the same time, the phospho-profile showed concurrent engagement of pro-survival and proliferative kinase pathways, including PI3K/Akt (Ser473) and MAPK/ERK (Thr202/Tyr204). This specific combination, concurrent DDR activation (p53-Ser15/Ser46, Chk2-Thr68, ATM-Ser1981) alongside pro-survival kinase engagement (Akt-Ser473, ERK-Thr202/Tyr204) and inflammatory signaling (RelA/p65-Ser529), defines a damage-tolerant survival state that, to our knowledge, has not been simultaneously characterized by integrated transcriptomics and phosphokinase arrays under chronic, low-dose BaP conditions. Mechanistically, PI3K/Akt/JNK axes have been linked to BaP-driven transcriptional activation [61], and our data position these pathways within a comprehensive p53/NF-κB-centered regulatory network that operates under sustained carcinogen pressure, enabling cellular persistence despite accumulated genotoxic burden.

Functional assays revealed that the chronic BaP phenotype is preneoplastic rather than fully transformative, and the negative transformation results are mechanistically informative. Neither clonogenic capacity nor anchorage-independent colony formation in soft agar were significantly altered after 12 weeks of BaP exposure at 0.1 or 1.0 µM (Kruskal–Wallis/Dunn test, *p* > 0.05 for all comparisons). The absence of anchorage-independent growth is particularly meaningful since anoikis resistance and anchorage-independent survival are hallmark features of malignant transformation [62], their absence under our chronic BaP conditions defines an important phenotypic boundary between the preneoplastic and fully transformed states. Furthermore, no tumor formation was observed in a pilot in vivo NOD/SCID assay, further supporting the conclusion that 12-week BaP exposure at environmentally relevant concentrations does not produce full malignant conversion. Together, these data, in the context of the strong molecular and histological alterations described above, delineate a mechanistic continuum in which chronic BaP exposure drives preneoplastic reprogramming that is necessary but not sufficient for malignant transformation, providing a reproducible experimental window to study early carcinogenesis independently of endpoint tumor formation.

The organotypic ALI model strengthened this interpretation by providing a tissue-scale endpoint that is central to early neoplastic evolution. Cultures derived from vehicle-treated cells formed a stratified, organized epithelium with normal apical-basal polarity, whereas cultures derived from 1.0 µM BaP-exposed cells displayed unequivocal dysplastic features by H&E histology: cellular atypia, nuclear pleomorphism, disruption of stratification, and loss of apical-basal polarity. This constitutes a histopathologically defined preneoplastic tissue phenotype directly caused by chronic BaP exposure, which according to our knowledge, has not been previously described in ALI cultures of the BEAS-2B model under chronic and low-dose conditions. These architectural changes are difficult to infer from 2D assays alone and underscore the value of incorporating a New Approach Methodology (NAM) that reports on epithelial organization and differentiation [63]. The coupling of 2D transcriptomic/phospho kinase array profiling with 3D organotypic tissue phenotyping in a single integrated platform represents a methodological advance for respiratory toxicology, enabling simultaneous mechanistic dissection and tissue-level validation of carcinogen-driven reprogramming. The dose-dependent PCNA patterns further suggest that proliferation and checkpoint control are spatially reprogrammed in chronic exposure states, potentially reflecting distinct balances of adaptation at 0.1 µM versus sustained checkpoint pressure at 1.0 µM.

Collectively, our results support a working model in which chronic BaP exposure drives an early, initiation-like adaptive state defined by: (i) sustained metabolic activation (CYP1A1/CYP1B1 induction at 4 h and 12 weeks); (ii) persistent DDR signaling (ATM-Ser1981, γH2AX, p53-Ser15/Ser46, Chk2-Thr68 phosphorylation); (iii) dose-dependent transcriptional remodeling away from ECM/adhesion programs and toward inflammatory and calcium-regulated modules (119 DEGs at 0.1 µM, 255 DEGs at 1.0 µM); and (iv) concurrent engagement of pro-survival kinase signaling (PI3K/Akt-Ser473, MAPK/ERK-Thr202/Tyr204) alongside NF-κB inflammatory activation (RelA/p65-Ser529). In this configuration, cells remain viable and metabolically active while tolerating ongoing genotoxic stress, a damage-tolerant survival state, without crossing the threshold into full malignant transformation. The molecular signature is instead manifested at the tissue scale as dysplastic ALI architecture, providing a coherent mechanistic bridge between carcinogen exposure, signaling network remodeling, and histological preneoplasia. This integrated characterization across genomic, phospho-kinase/NF-κB arrays, and organotypic dimensions establishes a multi-scale mechanistic framework for BaP-driven lung carcinogenesis that surpasses the resolution of previous single-endpoint or acute-exposure models.

Several limitations of the present study should be acknowledged. First, BEAS-2B cells are immortalized via the Ad12-SV40 hybrid virus and carry viral oncoproteins that may alter baseline p53 and cell-cycle signaling, potentially modifying the cellular response to BaP relative to primary bronchial epithelial cells; therefore, key transcriptional and signaling findings should be independently validated in normal human bronchial epithelial (NHBE) cells and donor-derived ALI cultures. The partial impairment of p53 function by SV40 LTAg means that the “damage-tolerant survival state” and the designation of p53 as a central regulatory hub described here should be interpreted cautiously. Primary cells with intact p53 might respond to equivalent chronic genotoxic stress with more pronounced apoptosis or senescence rather than adaptation, potentially leading to an overestimate of cellular survival capacity under chronic carcinogen pressure. This is a recognized limitation of SV40-immortalized models for studying p53-dependent carcinogenesis and has been noted elsewhere in the literature [64,65]. Second, we did not directly quantify BaP-DNA adduct levels (e.g., BPDE-N^2^-dG adducts by ^32^P-postlabeling or LC-MS/MS) or somatic mutation burden (e.g., by whole-exome sequencing), which would strengthen the mechanistic link between sustained DDR signaling and genomic instability and are necessary to define the mutagenic yield of the chronic exposure model. Third, the RNA-seq analysis was performed on bulk cell populations, precluding the identification of transcriptionally distinct subpopulations or clonal dynamics that may underlie the observed heterogeneity in DDR and proliferative responses. Thus, single-cell RNA-seq approaches would provide greater resolution. Fourth, the use of serum-containing (10% FBS) two-dimensional culture for chronic BaP exposure is standard practice but imposes baseline mesenchymal characteristics on BEAS-2B cells [26], which may complicate the interpretation of ECM/adhesion transcriptional changes that are among the most prominent DEGs identified. Serum-free or reduced-serum conditions, which more closely approximate the airway lumen environment, were not tested in this study; whether the observed transcriptional reprogramming is qualitatively maintained under such conditions remains to be determined in future studies. Finally, regarding causal inference, the regulatory network model centered on NF-κB, p53, and AP-1 is inferred from transcription factor–target interaction databases (TRRUST v2) and phospho-kinase/NF-κB array correlations. No gene silencing, inhibitor, or overexpression experiments were performed to establish causal relationships between these hubs and the observed phenotypic changes, remaining as an important priority for future work.

The findings of this study open several important avenues for future investigation. The dose-dependent transcriptional signatures identified here, particularly the repression of COL14A1, ADAMTS2, CSMD3, and CADM3, and the induction of NFATC4, SYT1, PCLO, and CSF2RA, constitute a candidate preneoplastic gene signature that warrants validation in primary human airway epithelial cells and, critically, in epidemiological or clinical cohorts of individuals with known BaP exposure or occupational PAH contact. Longitudinal transcriptomic and epigenomic profiling (e.g., ATAC-seq, DNA methylation arrays) at defined chronic exposure timepoints (4, 8, 12, 24 weeks) would clarify whether the observed reprogramming is progressive or exhibits phase transitions and would identify epigenetic regulatory mechanisms governing the biphasic transcriptional response documented here. Single-cell RNA-seq and clonal barcode tracing in the chronic BaP model would determine whether adaptation reflects reversible, stress-induced state transitions across the cell population, clonal selection of damage-tolerant subpopulations, or a combination of both, a fundamental open question in environmental carcinogenesis. The calcium-NFAT and vesicle-trafficking axis identified as upregulated under chronic BaP represents a novel and mechanistically unexplored component of the preneoplastic response, and its functional interrogation, using NFATC4 knockdown, calcium chelation, or secretome profiling, could reveal new therapeutic vulnerabilities in early carcinogenesis. Finally, integration of this model with CRISPR-based functional screens targeting the identified DEGs and phospho-kinase nodes would enable systematic causal assignment of molecular drivers of the preneoplastic phenotype, accelerating target prioritization for chemoprevention strategies.

In conclusion, this study establishes a mechanistic and operational framework linking chronic, environmentally relevant BaP exposure to a coherent preneoplastic program in human bronchial epithelial cells, validated across molecular, signaling, and tissue scales. By positioning this phenotype prior to malignant transformation, the integrated 2D/3D platform provides a reproducible experimental system for identifying the molecular determinants of early carcinogenesis and for evaluating chemoprevention strategies targeting the preneoplastic state, before the accumulation of irreversible oncogenic alterations that characterize overt lung malignancy.

## 5. Conclusions

Chronic sublethal BaP exposure (0.1–1.0 µM, 12 weeks) drives coherent preneoplastic reprogramming of BEAS-2B cells, combining sustained xenobiotic metabolic competence (CYP1A1/CYP1B1) with persistent genotoxic stress (ATM/γH2AX). Transcriptomic remodeling reveals repression of ECM/adhesion programs alongside activation of inflammatory and calcium-dependent signaling, converging on a p53-NF-κB/AP-1 regulatory nexus with concurrent PI3K/Akt and MAPK/ERK activation, consistent with a damage-tolerant survival state. These molecular changes translate into epithelial disorganization and dysplasia in organotypic ALI cultures, yet the absence of in vivo tumorigenicity supports an initiation/preneoplastic rather than fully malignant phenotype. This integrated 2D/3D platform provides a human-relevant framework for mechanistic dissection of PAH-driven lung carcinogenesis.

## Data Availability

The original contributions presented in this study are included in the article/supplementary material. Further inquiries can be directed to the corresponding author.

## Supplementary Materials

The following supporting information can be downloaded at: https://www.mdpi.com/article/10.3390/cells15060566/s1, Table S1: Primer sequences used for RT-qPCR and adapter sequences used for RNA-sequencing library preparation.

## Abbreviations

The following abbreviations are used in this manuscript:

AhR	Aryl hydrocarbon receptor
ALI	Air–Liquid Interface
ANOVA	Analysis of variance
ATCC	American Type Culture Collection
ATM	Ataxia-Telangiectasia Mutated
BaP	Benzo[a]pyrene
BEBM	Bronchial Epithelial Cell Growth Medium
BPDE	Benzo[a]pyrene-7,8-diol-9,10-epoxide
cDNA	Complementary DNA
CYP1A1	Cytochrome P450 Family 1 Subfamily A Member 1
CYP1B1	Cytochrome P450 Family 1 Subfamily B Member 1
DDR	DNA damage response
DEGs	Differentially expressed genes
DSBs	Double-strand breaks
ECM	Extracellular matrix
EMT	Epithelial–mesenchymal transition
FBS	Fetal bovine serum
GO	Gene Ontology
H&E	Hematoxylin and eosin
H2AX	H2A Histone Family Member X
IARC	International Agency for Research on Cancer
NAM	New Approach Methodology
PAH	Polycyclic aromatic hydrocarbon
Pan-CK	Pan-cytokeratin
PCNA	Proliferating Cell Nuclear Antigen
TF	Transcription factor
TRRUST	Transcriptional Regulatory Relationships Unraveled by Sentence-based Text-mining
γ-H2AX	P-H2AX (Ser139-P)
