# Supplementary material for: Modeling Chronic BaP Exposure in Bronchial Epithelial Cells Reveals Multi-Scale Drivers of Early Preneoplastic Reprogramming
Source: Cells. 2026 Mar 22;15(6):566. doi: 10.3390/cells15060566 (PMC13025689; doi:10.3390/cells15060566)
Supplement: Supplementary file 1 [file cells-15-00566-s001.zip › cells-4168630-supplementary.pdf]

Table S1. Primer sequences used for RT-qPCR and adapter sequences used for RNA-sequencing library preparation.

Gene	Primer Direction	Sequence (5′–3′)	Ta (°C)*
GAPDH	Forward	ATC AGC AAT GCC TCC TGC AC	61
	Reverse	ACA GTC TTC TGG GTG GCA GT	
CYP1A1	Forward	CCA GCT CAG CTC AGT ACC TC	60
	Reverse	CAT GGC CCT GGT GGA TTC TT	
CYP1B1	Forward	AAC AAG GAC CTG ACC AGC AG	60
	Reverse	CCC TGA AAT CGC ACT GGT GA	
MYC	Forward	TAC AAC ACC CGA GCA AGG AC	60
	Reverse	AGC TAA CGT TGA GGG GCA TC	
ZEB1	Forward	ACT GCC TGG TGA TGC TGA AA	60
	Reverse	CCC AAA CTG CAA GAA ACG CT	
ALX1	Forward	CAG GAC AGC AGC GTG AAC TA	60
	Reverse	CGG GAG ACA TTC GGA GAC TG	
SLC7A11	Forward	TCC ATG AAC GGT GGT GTG TT	60
	Reverse	TGG TAG AGG AGT GTG CTT GC	
PIR	Forward	TCA AAT TGG ACC CAG GAG CC	60
	Reverse	TCC AAG CAC TGC TGT GTG AT	
CCND1	Forward	GCG CAG ACC TTC GTT GCC CTC TG	68
	Reverse	CGG AGG CAG TCC GGG TCA CAC TT	
MGI adapters**	Forward	GCG CAG ACC TTC GTT GCC CTC TG	
	Reverse	CGG AGG CAG TCC GGG TCA CAC TT	

***Ta:** Annealing temperature

****MGI adapter sequences** utilized for the library construction
